# Particulate Matter Exposure and Preterm Birth: Estimates of U.S. Attributable Burden and Economic Costs

**DOI:** 10.1289/ehp.1510810

**Published:** 2016-03-29

**Authors:** Leonardo Trasande, Patrick Malecha, Teresa M. Attina

**Affiliations:** 1 Department of Pediatrics,; 2 Department of Environmental Medicine, and; 3 Department of Population Health, New York University (NYU) School of Medicine, New York, New York, USA; 4 NYU Wagner School of Public Service, New York, New York, USA; 5 NYU College of Global Public Health, New York, New York, USA

## Abstract

**Background:**

Preterm birth (PTB) rates (11.4% in 2013) in the United States remain high and are a substantial cause of morbidity. Studies of prenatal exposure have associated particulate matter ≤ 2.5 μm in diameter (PM2.5) and other ambient air pollutants with adverse birth outcomes; yet, to our knowledge, burden and costs of PM2.5-attributable PTB have not been estimated in the United States.

**Objectives:**

We aimed to estimate burden of PTB in the United States and economic costs attributable to PM2.5 exposure in 2010.

**Methods:**

Annual deciles of PM2.5 were obtained from the U.S. Environmental Protection Agency. We converted PTB odds ratio (OR), identified in a previous meta-analysis (1.15 per 10 μg/m3 for our base case, 1.07–1.16 for low- and high-end scenarios) to relative risk (RRs), to obtain an estimate that better represents the true relative risk. A reference level (RL) of 8.8 μg/m3 was applied. We then used the RR estimates and county-level PTB prevalence to quantify PM2.5-attributable PTB. Direct medical costs were obtained from the 2007 Institute of Medicine report, and lost economic productivity (LEP) was estimated using a meta-analysis of PTB-associated IQ loss, and well-established relationships of IQ loss with LEP. All costs were calculated using 2010 dollars.

**Results:**

An estimated 3.32% of PTBs nationally (corresponding to 15,808 PTBs) in 2010 could be attributed to PM2.5 (PM2.5 > 8.8 μg/m3). Attributable PTBs cost were estimated at $5.09 billion [sensitivity analysis (SA): $2.43–9.66 B], of which $760 million were spent for medical care (SA: $362 M–1.44 B). The estimated PM2.5 attributable fraction (AF) of PTB was highest in urban counties, with highest AFs in the Ohio Valley and the southern United States.

**Conclusions:**

PM2.5 may contribute substantially to burden and costs of PTB in the United States, and considerable health and economic benefits could be achieved through environmental regulatory interventions that reduce PM2.5 exposure in pregnancy.

**Citation:**

Trasande L, Malecha P, Attina TM. 2016. Particulate matter exposure and preterm birth: estimates of U.S. attributable burden and economic costs. Environ Health Perspect 124:1913–1918; http://dx.doi.org/10.1289/ehp.1510810

## Introduction

Preterm birth (PTB), defined as birth at < 37 weeks gestation, remains a substantial cause of early-life morbidity in the United States. In 2010, 35% of all infant deaths were attributable to preterm-related causes, with considerable disparities in rates across subpopulations (CDC 2014b). Preterm birth is recognized as a critical public health concern and, in addition, reflects persistent health disparities, being more prevalent among women of lower income status and from racial/ethnic minorities (Bryant et al. 2010). Although the national PTB rate in the United States has declined from a peak of 12.8% in 2006 to 11.4% in 2013, the rate of decline is currently insufficient to meet the March of Dimes goal of 5.5% by 2030 (McCabe et al. 2014). Reducing rates of PTB is important to prevent not only neonatal complications such as respiratory distress syndrome, sepsis, and intraventricular hemorrhage, but also adverse psychological, behavioral, and educational outcomes in later life, mostly related to cerebral palsy and neurodevelopmental delay (Saigal and Doyle 2008). In addition, preterm babies are at higher risk of developing systemic hypertension, diabetes, and stroke later in life (Norman 2013).

Although PTB and the often associated low birth weight (LBW; < 2,500 g) are highly multifactorial (with risk factors including maternal age, prenatal care, race, socioeconomic status, and preeclampsia) (Woodruff et al. 2009), most of these risk factors are not amenable to modification or avoidance. However, environmental factors, such as outdoor air pollutants, are amenable to change, through reductions in vehicular emissions, filtration of emissions from coal-fired power plants, and limits on residential use of coal and wood burning for heating and cooking (Trasande and Thurston 2005).

Though uncertainty remains about the contribution of specific outdoor air pollutants and windows of vulnerability, multiple observational studies of prenatal exposure have associated particulate matter ≤ 2.5 μm in diameter (PM_2.5_) among other pollutants with adverse birth outcomes, most especially LBW and PTB (Darrow et al. 2009; Kloog et al. 2012; Laurent et al. 2016), although some studies did not report this association (Johnson et al. 2016). In addition, one quasi-experimental study identified reductions in PTB and LBW in association with electronic toll collection, which also reduced traffic congestion and vehicle emissions.

Further support for the notion that outdoor air pollution exposure may contribute to adverse birth outcomes is provided by laboratory experiments that document oxidant stress, inflammation, and placental insufficiency as mechanisms by which air pollutants can contribute to early delivery (Institute of Medicine 2007; U.S. EPA 2013; Woodruff et al. 2009).

A major barrier to reductions in outdoor air pollution is the perception that these reductions will undermine economic productivity (Trasande et al. 2011). As with outdoor air pollution–associated respiratory illnesses, the costs associated with adverse birth outcomes are borne by society, rather than by those who gain from industrial processes that emit pollutants. These include health care costs for treatment of PTB-associated comorbidities and lost economic productivity due to PTB-associated reductions in cognitive potential. Yet, to our knowledge, estimates of the air pollution–attributable burden of PTB and associated economic costs have not been made. Our primary objective was to provide an estimate of the economic costs associated with PTBs attributable to PM_2.5_ exposure (as a proxy for outdoor air pollution) in the United States—estimates that could be used by decision makers when regulatory interventions to reduce air pollution exposures are considered.

## Methods

### General Approach

Outdoor air pollution is a complex mixture including, most notably, PM_2.5_ and PM_10_ (PM ≤ 10 μm), carbon monoxide, ozone, sulfur dioxide, and nitrogen dioxide. Recognizing that each of these air pollutants may independently contribute and/or modify the effects of exposure at an individual level, we used PM_2.5_ as a proxy for outdoor air pollution exposure, permitting use of a richer array of available measurements.

In the present analysis we adhered to the approach developed by the Institute of Medicine in assessing the “fractional contribution” of the environment to causation of illness, which is adapted below (Institute of Medicine 1981):

Attributable Costs = Anthropogenic increment in preterm birth incidence × Births × Cost per preterm birth

Because wildfires, dust storms, and volcanoes contribute to outdoor air pollution, reference levels (RL) were applied. We adopted an RL of 8.8 μg/m^3^, and incorporated a scenario with a 5.8-μg/m^3^ threshold in sensitivity analyses. We chose this approach because this was the reference level applied for other health effects of PM_2.5_ in the 2010 Global Burden of Disease estimates of PM_2.5_-attributable disease burden (i.e., the assumption was no health effects below this level) (Lim et al. 2012). Above the RL, all PM_2.5_ was considered of anthropogenic origin, and an attributable fraction (AF) of 100% was applied against the increment in cases and social costs of PTBs that could specifically be attributed to PM_2.5_.

Though studies have examined associations of outdoor air pollution with LBW (Woodruff et al. 2009), this association also presents additional complexity in attribution in that it has two major origins (PTB and intrauterine growth retardation, producing term LBW); therefore we limited our analysis to quantifying attributable PTB. In 2010, data from the U.S. Environmental Protection Agency (EPA) suggested that almost 124 million people in the United States lived in areas in which levels of some air pollutants were higher than the limits set by the National Ambient Air Quality Standards (Thorne 2016). Specifically, for PM_2.5_, the current limit set by the National Ambient Air Quality Standards is 12.0 μg/m^3^. Here we estimate effects of prenatal PM_2.5_ exposure on PTB occurring in the same year.

Subsequent sections describe approaches to estimating the PM_2.5_-attributable increment in PTB, the rate of prematurity, and costs.

### PM_2.5_-Attributable Fraction of Prematurity

We obtained daily averages of PM_2.5_ in 2008 for all ZIP codes in the conterminous United States as modeled by the EPA (U.S. EPA 2014). Recognizing that many counties have multiple ZIP codes, and ZIP codes may cross county boundaries, we estimated daily average PM_2.5_ for each county by averaging across all ZIP codes that constitute a portion of a given county. We then estimated deciles of PM_2.5_ exposure for each county in 2008. We assumed that births were evenly distributed over the course of the year, with 10% of births experiencing pregnancy-wide exposure to PM_2.5_ at the 90th percentile, and the next eight deciles of births exposed at the 80th, 70th, 60th, 50th, 40th, 30th, 20th, and 10th percentiles of PM_2.5_. The last decile of births was assumed to have no exposure or attributable PTB, whereas the other groups were assumed to have levels corresponding to the lowest extreme (e.g., 10th percentile for all exposure in the 10th–19th percentile grouping).

The most recent meta-analysis of English-language studies (Sapkota et al. 2012) estimated an odds ratio (OR) for PTB of 1.15 [95% confidence interval (CI): 1.14, 1.16] per 10 μg/m^3^ for pregnancy-wide exposure, and an OR of 1.07 (95% CI: 1.00, 1.15) per 10 μg/m^3^ for third-trimester exposure. In this meta-analysis, six studies contributed to the estimates for PM_2.5_ and PTB; only studies that examined PTB (< 37 weeks completed gestation) as the major end point and that reported results from single-pollutant models were included. Combined estimates of the OR were calculated based on data from all studies selected using fixed- and random-effects models; unlike for PM_10_, no significant heterogeneity was detected for studies that reported findings for PM_2.5_. Another recent study also identified an OR of 1.16 per 10 μg/m^3^ (see Figure S11 in Stieb et al. 2012) for the entire pregnancy (Stieb et al. 2012). We used the OR for pregnancy-wide exposure from the most recent meta-analysis (1.15 per 10 μg/m^3^) as the best estimate, but varied the OR from 1.07 to 1.16 in subsequent sensitivity analyses.

For each decile of births within each county, the OR from the meta-analysis was applied as a base with the exponent corresponding to the increment of the county-specific average PM_2.5_ above the assumed RL (8.8 μg/m^3^). The calculation is also depicted in the following formula:

OR_county-decile_ = OR_meta-analysis_^(decile of county-averaged PM_2.5_ – RL)/10 μg/m^3^^

Given that OR can overestimate relative risk (RR) and attributable fractions for common conditions such as PTB, we applied the formula described for estimating RR from OR and prevalence of PTB (Zhang and Yu 1998). For this calculation, county-level PTB rates for 2010 were obtained from the Centers for Disease Control and Prevention (CDC) WONDER database (CDC 2014a). Below is a numeric example to illustrate how RR was derived for Autauga County, Alabama.

First, we calculated the corresponding OR for each decile of exposure above the lowest decile. For example, using an OR of 1.15 (base case) for a 10-μg/m^3^ increase in PM_2.5_ and an RL of 8.8 μg/m^3^, the OR for the decile of births with the highest PM_2.5_ exposure would be estimated as

OR = 1.15 ^[(19.35 – 8.8)/10]^ = 1.16,

where 19.35 μg/m^3^ is the PM_2.5_ concentration of the 90th percentile of the distribution for the county. Similarly, for the PM_2.5_ concentration related to second lowest decile (20th) of average daily exposure for Autauga County (8.06 μg/m^3^),

OR = 1.15 ^[(8.06 – 8.8)/10]^ = 1.00.

Because this decile has PM_2.5_ concentration less than the RL, the corresponding estimated OR of 1 indicates no increase in risk and, therefore, the corresponding decile of births is assumed to have no PTBs attributable to PM_2.5_.

After deriving ORs for each decile, we use the formula by Zhang and Yu (1998) to estimate the RR for each decile, such that

RR = 1.16/[(1 – 0.15) + (0.15 × 1.16)] = 1.13,

where 0.15 is the PTB rate in that specific county. The range of RRs derived using these calculations was 1.06–1.18 (low–high scenarios).

Next, we computed the attributable fraction of PTB for outdoor air pollution for each decile, using the formula published by Levin (1953):

AF_PM2.5, county-decile_ = Prevalence_PM2.5exposure_ × (RR_county-decile_ – 1)/ [1 + Prevalence_PM2.5exposure_ × (RR_county-decile_ – 1)],

where the exposure prevalence is set to 10% for each decile. For example, for the highest decile of exposure in Autauga County, AF = [0.1 × (1.13 – 1)]/{1 + [0.1 × (1.13 – 1)]} = 0.013. Finally, we summed the AF for each decile (e.g., for Autauga County, the sum was 0.04), and multiplied the resulting value by estimated number of preterm births for each county, as shown below:

Cases of preterm births attributable to PM_2.5_ = 0.04 × 99.6 = 3.96, where 99.6 is the estimated number of preterm births in Autauga County. When aggregating AFs for each decile, a value of zero was assigned for AFs related to PM_2.5_ deciles below the RL (8.8 μg/m^3^), so that no cases of PTBs were attributed to those deciles of exposure.

### Population at Risk

Births in each county were obtained from the CDC WONDER database, as were county-level PTB rates (CDC 2014a), and multiplied together to calculate the number of preterm births in a county in 2010. For counties with population < 100,000, we estimated the number of births by taking the reported number of births across all these counties in each state and multiplying by the ratio of the county population to the total population of all counties with population < 100,000 from U.S. Census data (U.S. Census Bureau 2010). In addition, for these counties, the overall PTB rate of 0.15 was applied. The number of preterm births in each county was multiplied by the AF for each county to estimate the number of PM_2.5_-attributable premature births in 2010. Attributable PTBs in each county were aggregated to generate national estimates of attributable PTB.

### Estimates of PM_2.5_-Attributable Social Costs

Two direct costs of PTB were estimated: costs for treatment of PTB-associated medical conditions in the first 5 years of life, and costs after the first 5 years of life due to PTB-associated developmental disability. The direct health care costs in the first 5 years of life (estimated at $31,920) were obtained from the Institute of Medicine (2007) report on premature birth. Costs were also updated to 2010 dollars using the Medical Care Consumer Price Index (U.S. Department of Labor, Bureau of Labor Statistics 2014), and discounted 5 years at 3%/year to account for the expenses’ occurrence in the future. A similar approach was taken with health care costs after the first 5 years of life due to PTB-associated developmental disability (estimated at $1,920), except they were discounted by 3% for 15 years due to their occurrence further in the future.

Lost economic productivity due to reduced cognitive potential was also measured as an indirect cost of PM_2.5_-attributable PTB. PTB-associated IQ loss was calculated, using data from a systematic review that estimates an 11.9-point IQ decrement on average in PTB children (95% CI: 10.5, 13.4) (Kerr-Wilson et al. 2012). The loss in IQ was estimated by multiplying attributable PTB by the 11.9 IQ decrement, and the lost lifetime economic productivity was estimated by multiplying the IQ loss by 2%, which corresponds to the base case (range, 1.76–2.39%) described by Grosse et al. (2002), also used in previously published analyses (Trasande and Liu 2011), and the lifetime earnings estimate for a child born in 2010 (Max W, University of California, San Francisco; unpublished data, 2013). As with direct costs, all indirect costs are presented in 2010 dollars.

### Sensitivity Analyses

Recognizing uncertainty in the exposure–outcome relationship and RL, we performed sensitivity analyses. We used a range of ORs corresponding to different pregnancy-wide and third trimester–specific ORs (1.07–1.16) identified by the meta-analyses examining air pollution-PTB associations (Sapkota et al. 2012; Stieb et al. 2012) to represent sensitivity of the model to the nature of the exposure–outcome relationship. An alternative scenario with RL of 5.8 μg/m^3^ was also examined.

## Results

We examined 3,963,694 live births in the 48 contiguous United States, of which 475,368 (12%) were preterm births (Table 1). Applying the base case OR of 1.15 per 10-μg/m^3^ increment in PM_2.5_ and the reference level of 8.8 μg/m^3^, a median RR of 1.0031 was identified (IQR = 1.000–1.045; minimum, 1.00, maximum, 1.35) with 63.4% of births having RR > 1 (Table 2). We estimated that, across the 48 states, 3.32% of all preterm births in 2010 were attributable to PM_2.5_ (15,808; sensitivity analysis using ORs of 1.07 and 1.16: range, 7,532–29,968). These estimated numbers of attributable preterm births cost $760 million in medical care (sensitivity analysis: $362 million–1.44 billion), and $4.33 billion (sensitivity analysis: $2.06–8.22 billion) in lost economic productivity was also identified (based on estimated reductions in IQ and estimated consequences for productivity over a lifetime). In total, we estimated that $5.09 billion in PTB-related costs (medical care costs and lost economic productivity combined) could be attributed to PM_2.5_, with the sensitivity analysis producing a range in those costs of $2.43–9.66 billion.

**Table 1:** Live births and preterm births in the 48 U.S. states examined.

Parameter	Value
Total births, 48 contiguous U.S. states, 2010 (*n*)	3,963,694
Preterm births, 48 contiguous U.S. states, 2010 [*n* (%)]	475,368 (12.0)
Reference level, base case (sensitivity analysis)	8.8 μg/m^3^ (5.8)^*a*^
Odds ratio per 10 μg/m^3^ above reference level (sensitivity analysis)	1.15 (1.07, 1.16)
^***a***^For sensitivity analysis, a scenario with a 5.8-μg/m^3^ threshold was also used, above which all PM_2.5_ was considered of anthropogenic origin and an environmentally attributable fraction of 100% was applied.	

**Table 2:** Estimated economic costs of PM_2.5_-attributable preterm births.

Parameter	Base scenario^*a*^	Low scenario^*a*^	High scenario^*a*^
Range of relative risks	1.000–1.352	1.000–1.159	1.000–1.429
Median relative risk (IQR)^*b*^	1.003 (1.000–1.045)	1.002 (1.000–1.021)	1.043 (1.011–1.089)
Percentage with RR above 1 (RR = 1 indicates risk unchanged)	63.4%	63.4%	91.7%
Attributable fraction	3.32%	1.58%	6.30%
Attributable preterm births (*n*)	15,808	7,532	29,968
Lost economic productivity, PM_2.5_-attributable preterm births^*c*^	$4.33 billion	$2.06 billion	$8.22 billion
Additional medical care, PM_2.5_-attributable preterm births^*d*^	$760 million	$362 million	$1.44 billion
Total costs, PM_2.5_-attributable preterm births	$5.09 billion	$2.43 billion	$9.66 billion
IQR, interquartile range. ^***a***^Base scenario estimates are based on OR of 1.15; low and high scenario estimates are based on ORs of 1.07 and 1.16, respectively. For calculations, please see “Methods.” ^***b***^Median RR estimated using the MEDIAN function in Excel (Microsoft). ^***c***^Lost economic productivity due to reduced cognitive potential was measured as an indirect cost of PM_2.5_-attributable PTB. PTB-associated IQ loss was calculated using data from a systematic review that estimates an 11.9-point IQ decrement on average in PTB children (95% CI: 10.5, 13.4). See “Methods.” ^***d***^Two types of direct costs of PTB were estimated: costs for treatment of PTB-associated medical conditions in the first 5 years of life, and costs after the first 5 years of life due to PTB-associated developmental disability. See “Methods.”			

Substantial variability in estimated attributable fraction and preterm births was identified at the state level in base case analyses. In Ohio, the attributable fraction was highest (5.44%), whereas only 0.12% of preterm births were attributable to PM_2.5_ in New Mexico and Wyoming. California had the largest number of attributable preterm births (2,149) and costs ($692 million) in base case analyses (Table 3).

**Table 3:** Results by state for a 10-μg/m^3^ increment in PM_2.5_ above the reference level of 8.8 μg/m^3^ (base case estimates).

State	Estimated attributable fraction (%)	Estimated attributable preterm births (*n*)	Estimated attributable lost lifetime economic productivity	Estimated attributable medical care costs
Alabama	4.31	404	$110 million	$19.4 million
Arizona	0.58	61	$16.8 million	$2.95 million
Arkansas	3.18	156	$42.8 million	$7.50 million
California	4.27	2,149	$589 million	$103 million
Colorado	0.43	31	$8.50 million	$1.49 million
Connecticut	2.87	112	$30.6 million	$5.36 million
Delaware	4.70	68	$18.8 million	$3.29 million
Florida	0.87	249	$68.4 million	$12.0 million
Georgia	5.17	950	$260 million	$45.7 million
Idaho	0.90	22	$5.92 million	$1.04 million
Illinois	4.87	976	$268 million	$46.9 million
Indiana	5.40	532	$146 million	$25.6 million
Iowa	2.94	132	$36.1 million	$6.32 million
Kansas	2.63	113	$31.1 million	$5.44 million
Kentucky	4.62	354	$97.1 million	$17.0 million
Louisiana	2.32	218	$59.8 million	$10.5 million
Maine	0.85	11	$2.94 million	$515,000
Maryland	4.67	438	$120 million	$21.1 million
Massachusetts	2.44	190	$52.2 million	$9.15 million
Michigan	3.81	533	$146 million	$25.6 million
Minnesota	2.46	172	$47.1 million	$8.26 million
Mississippi	2.65	187	$51.2 million	$8.97 million
Missouri	3.48	323	$88.5 million	$15.5 million
Montana	0.33	5	$1.31 million	$229,000
Nebraska	1.64	48	$13.3 million	$2.33 million
Nevada	0.57	28	$7.76 million	$1.36 million
New Hampshire	1.61	19	$5.31 million	$931,000
New Jersey	3.95	490	$134 million	$23.6 million
New Mexico	0.12	4	$1.05 million	$185,000
New York	3.67	1,032	$283 million	$49.6 million
North Carolina	4.23	658	$181 million	$31.6 million
North Dakota	0.44	4	$1.21 million	$211,000
Ohio	5.44	924	$253 million	$44.4 million
Oklahoma	2.47	182	$50.0 million	$8.77 million
Oregon	1.63	74	$20.2 million	$3.55 million
Pennsylvania	5.04	819	$224 million	$39.4 million
Rhode Island	1.99	24	$6.59 million	$1.16 million
South Carolina	3.88	321	$87.9 million	$15.4 million
South Dakota	0.87	12	$3.21 million	$563,000
Tennessee	4.17	425	$116 million	$20.4 million
Texas	2.47	1,251	$342 million	$60.1 million
Utah	1.70	97	$26.5 million	$4.65 million
Vermont	1.12	6	$1.61 million	$282,000
Virginia	3.71	444	$122 million	$21.4 million
Washington	1.12	98	$26.9 million	$4.71 million
West Virginia	4.62	114	$31.4 million	$5.50 million
Wisconsin	3.85	286	$78.4 million	$13.7 million
Wyoming	0.12	1	$264,000	$46,400
District of Columbia	4.73	59	$16.2 million	$2.84 million
Base case scenario refers to OR of 1.15 per 10-μg/m^3^ increment in PM_2.5_ and the reference level of 8.8 μg/m^3^. Estimated attributable fraction: the fraction of PTBs attributable to outdoor air pollution. Estimated attributable preterm births: estimated number of PTBs attributable to outdoor pollution. Estimated attributable lost lifetime economic productivity: PTB-associated IQ loss resulting in lost economic productivity. Estimated attributable medical care costs: costs for treatment of PTB-associated medical conditions in the first 5 years of life and costs after the first 5 years of life due to PTB-associated developmental disability.				

**Figure 1:**
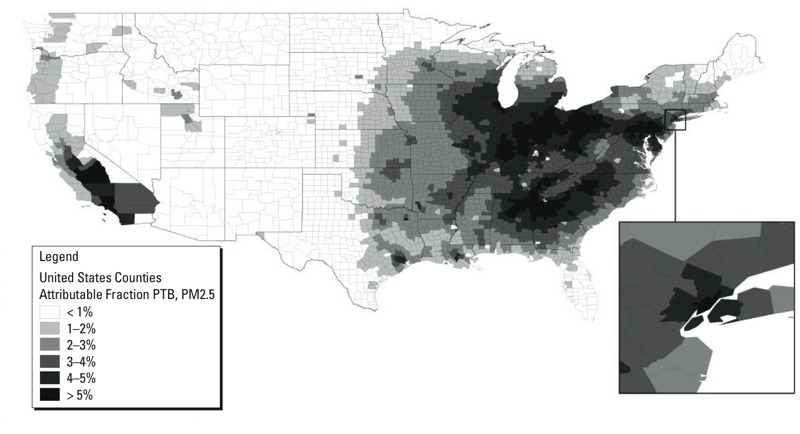
Fraction of preterm birth attributable to air pollution, county-level data. Births in each county were obtained from the CDC WONDER database (CDC 2014a), as were county-level PTB rates, and multiplied together to calculate the number of preterm births in a county in 2010. For counties with population < 100,000, the overall rate (0.15) for those counties was applied. The number of preterm births in each county was multiplied by the AF for each county to estimate the number of PM_2.5_-attributable premature births in 2010. Source for PM_2.5_ data: U.S. Environmental Protection Agency (U.S. EPA 2008).

At the county level, the variability in attributable preterm births was greater than at the state level, as presented in Figure 1 and detailed in Table 3, which indicates a range of 0.12–5.44%. The PM_2.5_ AF of PTB was generally higher in major urban regions. Consistent with the state level results, the highest AFs (> 5%) were identified in the Ohio valley, the southern United States, southern California, southeastern Pennsylvania, New York City, and Chicago, Illinois.

## Discussion

The main finding of this analysis suggests that exposure to PM_2.5_ contributes significantly to preterm birth in the United States and translates into substantial economic loss over a lifetime. To our knowledge, this is the first time that such economic estimates are reported, and these suggest that considerable health and economic benefits can be gained through reductions in outdoor air pollution exposure in pregnancy.

Exposure to PM_2.5_ has been associated with PTB in a number of studies, although with variable results, as highlighted in the meta-analysis by Stieb et al. (2012). This meta-analysis included 62 studies and reported substantial heterogeneity among studies, as well as variability in risk by gestational period. Of note, one quasi-experimental (not observational) study identified reductions in PTB and LBW in association with electronic toll collection, which also reduced traffic congestion and vehicle emissions (Currie and Walker 2011). Studies to date have applied different methodological approaches to exposure and outcome assessment, and have been conducted in many regions of the world where air pollution composition may vary, accounting for differential effects (Woodruff et al. 2009). This is especially true for PM_2.5_, which is a complex mixture of different chemicals, and may contribute to explaining the variable results obtained in these studies, with different mixtures leading to different outcomes (Harris et al. 2014), also depending on specific windows of exposure (Rappazzo et al. 2014). In addition, differences in population susceptibility most likely contribute to the observed variability.

We used 5.8–8.8 μg/m^3^ as reference levels, following the approach used by the 2010 Global Burden of Disease collaborators (Lim et al. 2012). These levels can be considered minimum achievable levels of PM_2.5_ insofar as anthropogenic sources can be limited, and no safe level of PM_2.5_ exposure has been identified. The estimated 15,808 preterm births can be considered preventable through strategies to reduce PM_2.5_ exposure, though future work can model reductions due to changes in vehicular and other emissions considering the impact of regulatory and other interventions as counterfactuals, so as to inform cost–benefit and other regulatory impact analyses.

In our analysis we estimated loss of IQ related to preterm birth and its impact on earning potential, which can be considered the result of direct effects, such as lower cognitive capacities, and indirect effects due to diminished educational achievements and reduced ability to work (Grosse et al. 2002). Estimating and aggregating at the national level the economic costs associated with PM_2.5_-attributable preterm births provide a sense of the potential economic benefits that could be achieved by regulatory interventions aimed at reducing air pollution exposure during pregnancy. In addition, long-term health issues associated with preterm birth that prevent individuals from working translates into increased government expenditures for programs such as Supplemental Security Income, further adding to the economic costs shouldered by society as a whole (Perrin et al. 2007). Last but not least, it is important to consider that PTB also places an important emotional and psychological burden on parents and families, which, although nonfinancial, needs to be taken into account when considering the benefits that could be achieved by such regulatory interventions.

### Limitations

There are important limitations to the interpretation of our findings. The specific components of outdoor air pollution that contribute to prematurity and other adverse birth outcomes remain elusive, as do the mechanisms by which they produce effects. Although it is true that some studies to date have failed to find significant associations with adverse outcomes, exposure imprecision may have biased those estimates (Fleiss and Shrout 1977); others may have had modest statistical power to detect significant differences in prematurity. Therefore, some may argue that the scientific evidence for air pollution has not reached the threshold for causation. We take heed of Sir Austin Bradford Hill’s landmark treatise on criteria for causation, in which he raises the need to consider the decision at hand in weighing the strength of the scientific evidence: “On fair evidence we might take action on what appears to be an occupational hazard…without too much injustice if we are wrong” (Hill 1965). More recently, we have developed methods to estimate probability of causation for diseases attributable to endocrine-disrupting chemicals (Trasande et al. 2015). Although the scope of the present analysis was limited and we did not formally evaluate the epidemiologic and toxicologic evidence, the evidence for air pollution and its effects on fetal growth are of similar strength to many of the exposure–outcome relationships considered in this more recent work. The evidence for causation of preterm births by outdoor air pollutants is strong, although not uniform, in that multiple observational studies have identified significant relationships, as evidenced by some of the most recent meta-analyses (Sapkota et al. 2012; Stieb et al. 2012). Though studies have not always yielded identical results, the findings in humans are consistent with those in the laboratory. These support the estimation of disease burden and costs as presented here, though we have not estimated a probability of causation as others have pursued in the presence of uncertainty (Trasande et al. 2015).

Models are also only as good as their inputs. Stationary-monitor sites do not take into account variation in personal exposure levels, variations in PM_2.5_ levels within the geographical area monitored by each site, or changes in residence during pregnancy. Data for ultrafine PM were not available, even though this form may pose a greater risk of adverse birth outcomes than PM_2.5_. PM_2.5_ measurements were estimated at the county level, and insofar as pregnancies are not evenly distributed by outdoor air pollution, our approach introduces some imprecision. We also estimated attributable PTB assuming homogeneity in the PTB rate across small population counties, which may have added imprecision in county-level estimates. We did not consider effects on maternal health, or possible stillbirths and birth defects that are plausible although less supported by the literature (Woodruff et al. 2009).

Air pollution exposures over the period January–December 2008 can be expected to influence a broad period of pregnancies spanning at least 9 months later, with birth dates spanning September 2008–August 2009 and most births in 2009. We acknowledge some imprecision in selecting the 2010 birth year to extrapolate disease burden. In part, the intention was to bring estimates in line with other environmentally related disease burden estimates that use 2010 as a base (Trasande et al. 2015), and ensure comparability. Our decision was supported by the relative lack of changes in mean PM_2.5_ between 2009 and 2010 (Thorne 2016).

We also examined PTB as a categorical outcome, when shifts in gestational age are more likely to occur due to PM_2.5_. Insofar as gestational age is not normally distributed, estimated PM_2.5_-induced shifts in gestational age may potentially result in larger effects on PTB than the estimates we modeled here. PTB data from CDC are also based on last menstrual period, when the definition is changing to clinically based obstetric estimates. A substantial literature suggests misclassification in both directions, with generally estimates based on last menstrual period producing higher PTB rates (Hall et al. 2014; Qin et al. 2007), so our methodology may have produced modestly higher estimates than those obtained using clinically derived PTB data.

## Conclusions

Our estimates suggest that PM_2.5_ may contribute substantially to the burden and costs of PTB in the United States. Because of the widespread exposure to PM_2.5_, considerable health and economic benefits could be achieved through regulatory interventions that reduce such exposure in pregnancy. Furthermore, the differential impact of air pollution by socioeconomic status and race/ethnicity, as suggested by Bryant et al. (2010), underscores the importance of regulatory actions aimed at reducing exposure, because these may also reduce the well-known and long-standing disparities in preterm births.
